# Robot-Assisted Laparoscopic Resection of a Transverse Colon Schwannoma

**DOI:** 10.1155/2020/8057352

**Published:** 2020-03-12

**Authors:** Miguel Rodriguez, Collin Stewart, Omar Khan, Brian Citro

**Affiliations:** ^1^HCA Sunrise Health GME Consortium, Department of Surgery, Las Vegas, NV, USA; ^2^LMC Pathology Services, Las Vegas, NV, USA

## 1. Introduction

Schwannomas are tumors that originate from the myelin sheath of Schwann cells [1]. These tumors are rare, benign, and slow-growing neoplasms that arise from peripheral nerves throughout the body, including the gastrointestinal tract [2]. Schwannomas are characterized by a well-circumscribed capsule, accounting for 2-6% of all gastrointestinal mesenchymal tumors. Other gastrointestinal mesenchymal tumors include smooth muscle cell tumors, neurofibromas, and gastrointestinal stromal tumors (GISTs) [3, 4]. Schwannomas are mostly seen during the third through fifth decades of life, with men and women affected equally. Due to the limited cases of gastrointestinal schwannomas in the literature, the characteristics of this tumor are yet to be defined [5]. We present a case of an asymptomatic 83-year-old female with a gastrointestinal schwannoma. The mass was found incidentally on abdominal/pelvic computed tomography (CT). Robotic-assisted laparoscopic resection was performed. This case is being presented as the incidence of gastrointestinal schwannomas is very low.

## 2. Case

**Figure 1:**
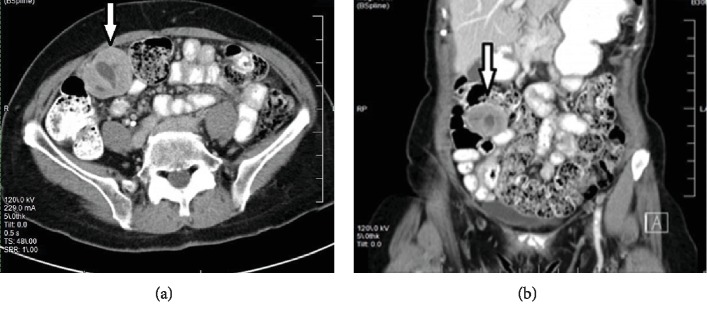
Axial (a) and coronal (b) views of CT scan of the abdomen and pelvis. Notice the right lower quadrant mass (white arrow) with low attenuation over the central region.

An 83-year-old female presented to our clinic for a mass over the right lower abdomen. She denied having symptoms such as abdominal pain, nausea, vomiting, or bowel changes. She had been diagnosed with this mass in 2015 by her primary care physician, but she failed to follow up. The patient's medical history was significant for hypertension and hysterectomy. There was no reported personal or family history of inflammatory bowel disease or gastrointestinal cancer. She reported no previous history of esophagogastroduodenoscopy or colonoscopy. On examination, patient was hemodynamically stable. Her abdomen was soft and nontender and had no gross palpable masses. Laboratory test results were within normal limits. A repeat CT of the abdomen and pelvis revealed a well-circumscribed mass anteriorly adjacent to the cecum and just deep to the intra-abdominal wall musculature, measuring 4.4 cm in transverse dimension and 4.0 cm in anteroposterior dimension, with low attenuation in the central region (Figure 1).

**Figure 2:**
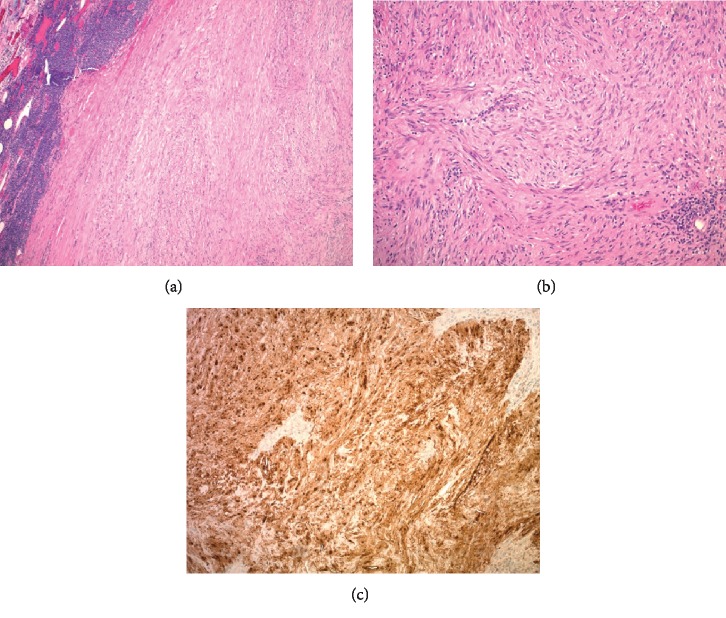
(a) Well-circumscribed spindle cell lesion with peripheral lymphoid aggregates (hematoxylin-eosin, original magnification ×4). (b) The lesion is moderately cellular composed of bland spindle cells within a neurofibrillary background (hematoxylin-eosin, original magnification ×10). (c) S-100 immunohistochemical study shows diffuse positivity (original magnification ×10).

There was no other abnormal pathology identified throughout the small and large intestines. CT-guided core needle biopsy revealed spindle cells under hematoxylin and eosin (H&E) sections (Figures 2(a) and 2(b)). Immunohistochemical analysis of these spindle cells was positive for S-100 and synaptophysin (Figure 2(c)). No reactivity for cluster of differentiation (CD) 20, CD3, pancytokeratin, and desmin was detected. Given the morphologic features (neurofibrillary background) and S-100 positivity, the diagnosis of a schwannoma was favored. CD117 (c-kit) staining of the specimen was not necessary to rule out GIST. GIST does not have strong S-100 positivity nor does it have neurofibrillary background.

A robot-assisted laparoscopic right hemicolectomy with an intracorporeal anastomosis was the procedure planned for the suspected cecal mass. A 5 mm Optiview technique was used over the left upper quadrant at Palmer's point to access the intra-abdominal cavity. The abdomen was insufflated to 15 mmHg. Two additional robotic ports were placed inferior to the initial port under direct visualization. These ports were separated by a distance of 8-10 cm. The da Vinci Xi surgical system was docked. Lysis of adhesions was performed in the pelvic region. Upon further dissection, it was noted that the suspected cecal mass was actually a transverse colon mass being held down by adhesions over the right lower quadrant. The operative approach was changed due to the intraoperative findings. A midline hand-assisted port incision was made and the transverse colon was eviscerated. A segmental resection with 5 cm margins was performed, followed by an extracorporeal side-to-side stapled anastomosis. Lembert sutures were used to reinforce the staple line. The colon was placed back into the abdominal cavity. The fascia and skin were closed. The patient tolerated the procedure well without complications. She was discharged home on postoperative day one after appropriate pain control, food tolerance, and adequate ambulation.

The operative specimen was grossly described as a discrete well-circumscribed encapsulated mass focally abutting the underlying mucosa. There was no involvement of the mucosal surface, and there was no extension through the intestinal wall.

## 3. Discussion

Primary gastrointestinal schwannomas are rare unless there is an association with neurofibromatosis (i.e., von Recklinghausen disease). Although generally asymptomatic, these tumors may cause symptoms such as abdominal pain, rectal pain, hematochezia, and tenesmus [6]. Both imaging and endoscopic procedures play an important role in the initial diagnosis of schwannomas. However, immunohistochemical analysis is the gold standard diagnostic test. Immunoexpression of S-100 protein with low affinity for CD34, cytokeratins, and desmin suggests the diagnosis of schwannoma [6]. The biologic behavior of schwannomas is not fully understood; therefore, no established parameters predictive of malignancy exist. Mitotic activity (>5 mitoses per high-power field) and tumor size (>5 cm) are two factors that clinicians rely on to predict malignancy [7]. Wide excision and extensive lymphadenectomy are not recommended as it has been found that schwannomas carry a low risk of malignancy [8]. A robotic approach was chosen for our case as there is evidence that robotic right hemicolectomy has lower estimated blood losses, shorter hospital stays, lower rates of overall complications, and a significantly faster bowel function recovery compared to laparoscopic right hemicolectomy [9]. On another meta-analysis, conversion to open surgery was more common during laparoscopic approach compared to robotic approach [5]. Two disadvantages of robotic surgery are higher costs and longer operative time. In our opinion, both of these disadvantages are offset by the advantages. We cannot make an argument in favor of laparoscopic right hemicolectomy knowing that a patient may have more complications and a longer hospital stay. These may add higher costs, which could be more than the robotic approach itself. In our institution, we have dedicated robotic surgical teams with extensive training and experience, which increases efficacy of the procedure. We are unable to comment on the robotic ergonomic advantages on this case as the initial procedure planned was not performed. In theory, the three-dimensional view and the endowrist movements of the robot would have facilitated the mobilization of the colon and the intracorporeal anastomosis. The evidence on the use of chemotherapy or radiotherapy has not been defined for gastrointestinal schwannomas; therefore, it is not recommended routinely. Postoperative surveillance should be considered for aggressive tumors [6]. This case was presented due to the rarity of gastrointestinal schwannomas. Furthermore, we support the use of robotic surgery on colon cases.
